# Distribution of *Candida* Species Causing Oral Candidiasis in High-Risk Populations: A Systematic Review

**DOI:** 10.3390/healthcare14020159

**Published:** 2026-01-08

**Authors:** João Pedro Carvalho, Jéssica Rodrigues, Célia Fortuna Rodrigues, José Carlos Andrade, António Rajão

**Affiliations:** 1 UNIPRO—Oral Pathology and Rehabilitation Research Unit, University Institute of Health Science (IUCS), 4585-116 Gandra, Portugal; a29614@alunos.cespu.pt (J.P.C.); a27019@alunos.cespu.pt (J.R.); 2 Associate Laboratory i4HB—Institute for Health and Bioeconomy, University Institute of Health Sciences—CESPU, 4585-116 Gandra, Portugal; celia.fortuna@iucs.cespu.pt (C.F.R.); jose.andrade@iucs.cespu.pt (J.C.A.); 3 UCIBIO—Applied Molecular Biosciences Unit, Translational Toxicology Research Laboratory, University Institute of Health Sciences (1H-TOXRUN, IUCS-CESPU), 4585-116 Gandra, Portugal; 4 LEPABE—Laboratory for Process Engineering, Environment, Biotechnology and Energy, Faculty of Engineering, University of Porto, 4200-465 Porto, Portugal; 5 ALiCE—Associate Laboratory in Chemical Engineering, Faculty of Engineering, University of Porto, 4200-465 Porto, Portugal

**Keywords:** *Candida* spp., oral candidiasis, oral isolates

## Abstract

**Background:** In the last decade, infections caused by *Candida* species have increased. Although *C. albicans* remains the most predominant species, fungal infections caused by non-*albicans Candida* (NAC) species have also been rising. This study aimed to determine which *Candida* spp. are most frequently associated with oral candidiasis. **Methods:** In accordance with PRISMA guidelines, a literature search was conducted in the PubMed, Cochrane Library, and ScienceDirect databases. The search used the keyword combination “candida spp” AND “oral candidiasis” AND “oral isolates” and included articles published between 2013 and 31 October 2025. **Results:** A total of 658 articles were identified, of which 24 met the inclusion criteria. Across these studies, 12,750 isolates were reported. *C. albicans* was the most prevalent species, accounting for 81.7% of all isolates. NAC species were detected at lower frequencies, including *C. tropicalis* (7.2%), *C. glabrata* (4.5%), *C. krusei* (4.1%), *C. parapsilosis* (1.0%), *C. dubliniensis* (0.8%), *C. kefyr* (0.2%), *C. guilliermondii* (0.1%), *C. lusitaniae* (0.1%), and other rare or unidentified species (0.2%). The increasing prevalence of *Candida* infections is associated with a growing population of immunocompromised individuals, and treatment remains challenging due to rising antifungal resistance. **Conclusions:** Although *C. albicans* remains the most prevalent, the appearance of NAC species is gradually increasing. With the increase of *Candida* spp. resistant to conventional antifungal agents and with the competitive or synergistic interaction between *Candida* spp., it is necessary to develop new therapeutic approaches.

## 1. Introduction

Several *Candida* spp. are commonly found in the oral cavity and constitute part of the normal microbiota in healthy individuals. However, these organisms are opportunistic commensals that, under certain predisposing conditions, can become pathogenic and cause infections in the oral cavity, collectively known as oral candidiasis [1,2,3,4,5,6,7].

In recent years, infections caused by *Candida* spp. have risen markedly, largely due to the growing population of immunocompromised individuals. This includes patients with diabetes, human immunodeficiency virus (HIV) infection, or malignancies, as well as those who smoke, have fissured tongues, or use removable dentures or orthodontic appliances. Treating fungal infections presents a significant challenge in clinical practice, mainly because of the increasing resistance of *Candida* spp. to antifungal therapies, particularly azoles such as fluconazole and itraconazole, and polyenes such as nystatin [1,2,3,4,5,6,7,8,9,10,11,12].

Although *C. albicans* remains the most prevalent etiological agent, the incidence of infections caused by NAC species has been increasing. For example, *C. dubliniensis* shares close phenotypic similarities with *C. albicans* but may exhibit greater virulence, as evidenced by its higher proteinase activity and enhanced ability to adhere to oral epithelial cells. Similarly, *C. glabrata* displays intrinsic resistance to azoles—the most frequently used topical agents for biofilm-associated fungal infections—making oral mucosal infections caused by this species particularly difficult to manage. Furthermore, newly emerging *Candida* spp. continue to pose challenges to the development of effective antifungal treatments [7,8,9].

Several factors contribute to the growing antifungal resistance observed among *Candida* spp., including global demographic changes, an aging population, cancer therapies, and prolonged use of antifungal drugs [2,6,11]. Poor oral hygiene, xerostomia, prosthetic or orthodontic devices, immunosuppression, prior head and neck radiotherapy, smoking, and alcohol use increase the risk of oral candidiasis. Other risk factors include corticosteroid therapy, poor glycemic control, increased enzymatic activity, reduced salivary pH, and impaired tissue response to injury [5,6,10,11,13,14].

Nutritional deficiencies and metabolic disturbances also play a role in the development of oral candidiasis. Conditions such as malnutrition, malabsorption syndromes, eating disorders, high-carbohydrate diets, and deficiencies in iron, zinc, magnesium, selenium, folic acid, and vitamins A, B6, B12, and C have all been implicated as contributing factors [13,14].

Clinically, oral candidiasis manifests through various signs and symptoms, including white or yellow plaques, oral ulcers, dysgeusia, dysphagia, odynophagia, and glossodynia. The infection may present in several distinct clinical forms, including pseudomembranous, erythematous, chronic hyperplastic, denture stomatitis, and angular cheilitis, each exhibiting characteristic clinical features [2,6,14,15].

Pseudomembranous candidiasis, the most common variant, primarily affects the tongue and oral mucosa. It typically occurs in infants and patients with chronic illnesses and is characterized by soft, elevated white plaques composed of desquamated epithelium, necrotic debris, keratin, leukocytes, fibrin, and bacteria. Upon removal of these plaques, erythematous, inflamed mucosal areas are often revealed [2,16].

Erythematous candidiasis usually arises following prolonged use of antibiotics or corticosteroids and presents as painful erythematous lesions (without white plaques), most frequently involving the palate and tongue, often accompanied by central papillary atrophy [2].

Chronic hyperplastic candidiasis, also known as candidal leukoplakia, is characterized by white plaques that cannot be scraped off. These lesions, which may affect the lips, tongue, or buccal mucosa, can appear homogeneous or nodular and are sometimes considered potentially malignant [2,17,18].

The diversity of oral *Candida* species and increasing antifungal resistance underscore the need for improved diagnostic methods, which are essential for the timely implementation of targeted therapeutic strategies [6,9,13].

Therefore, this systematic review aims to identify the *Candida* spp. most commonly associated with oral candidiasis infections, thereby contributing to improved diagnostic precision and more effective clinical management of this condition.

## 2. Materials and Methods

### 2.1. Review Guidelines

This systematic review was conducted in accordance with the Preferred Reporting Items for Systematic Review and Meta-analysis (PRISMA) 2020 guidelines [19]. The study protocol was registered in the PROSPERO database (CRD420251236585).

### 2.2. Selection Criteria

To be included in this study, articles had to meet the following conditions:

Inclusion criteria:

- Articles published between 2013 and 31 October 2025;
- Studies involving human participants clinically diagnosed with oral candidiasis;
- Studies identifying, isolating, or characterizing *Candida* spp. obtained from the oral cavity;
- Original research studies including cohort (prospective or retrospective), cross-sectional, case-control, or longitudinal studies.

Exclusion criteria:

- Articles that did not address oral candidiasis or *Candida* spp.;
- Systematic or narrative reviews, meta-analyses, case reports, conference abstracts, books, or other non-peer-reviewed documents.

### 2.3. Eligibility Criteria

The research question was formulated using the PICO (Population, Intervention, Comparison, and Outcomes) strategy: “Among individuals with oral candidiasis, which *Candida* species are most frequently identified as causative agents?” (Table 1).

### 2.4. Search Strategy

A comprehensive literature search was conducted in PubMed, Cochrane Library, and ScienceDirect for studies published between January 2013 and 31 October 2025. In ScienceDirect, in addition to the time limit, the filter for “Research Articles” was applied. Various combinations of keywords were applied to identify relevant scientific articles in accordance with the objectives of this study. The specific keywords used in the search strategy are summarized in Table 2.

### 2.5. Selection of Articles and Data Collection

To reduce selection and data extraction bias, two reviewers (J.R. and J.P.C.) independently screened all retrieved records and performed data extraction in duplicate. Disagreements were resolved through discussion, and when consensus could not be achieved, a third reviewer (A.R.) adjudicated. This procedure ensured inter-rater reliability and methodological rigor. The literature search was conducted electronically, and all records retrieved from the databases were exported to Mendeley Reference Manager^®^, where duplicate entries were automatically identified and removed.

### 2.6. Quality Assessment and Risk of Bias

The methodological quality and risk of bias of the included studies were assessed using the Joanna Briggs Institute (JBI) Critical Appraisal Checklists: the Checklist for Cross-Sectional Studies, the Checklist for Cohort Studies, and the Checklist for Case-Control Studies [20]. Each item in Table 3 was rated as Yes (Y), No (N), Unclear (U), or Not Applicable (NA). The risk of bias assessment was independently performed by two reviewers (J.P.C. and J.R.), and any disagreements were resolved through discussion and consensus with a third reviewer (A.R.). Four studies were assessed as having a low risk of bias, while twenty studies were classified as having a moderate risk of bias.

## 3. Results

### 3.1. Selection of Articles

**Figure 1:**
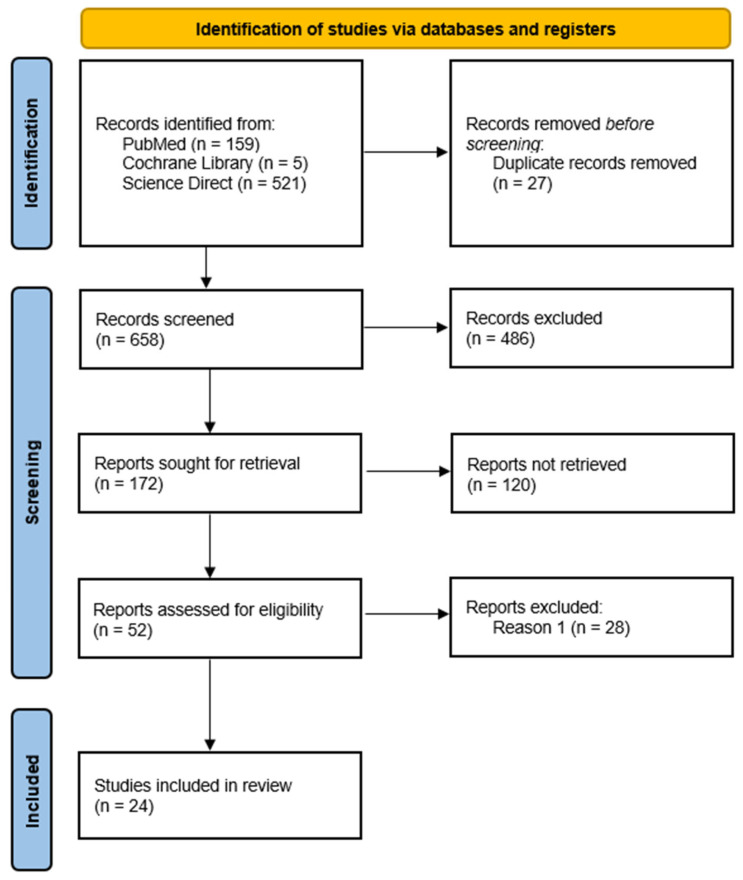
PRISMA flowchart of the studies identified through electronic search. Reason 1: Irrelevant to the topic.

Searches of the PubMed, Cochrane Library, and ScienceDirect databases yielded a total of 685 records. After the removal of 27 duplicates, 658 records remained for screening. Titles and abstracts were then assessed, and the eligible articles underwent full-text review. Ultimately, 24 studies met the inclusion criteria and were included in this study, as shown in Figure 1.

### 3.2. Sample Characteristics for Study Quality

For the cross-sectional studies, 14 studies showed a moderate risk of bias: Sanitá et al. [21], 2014, Muadcheingka et al. [22], 2015, Prakash et al. [25], 2015, Mohammadi et al. [27], 2016, Lourenço et al. [28], 2017, Portela et al. [29], 2017, Castillo et al., 2018 [30], Spalanzani et al. [31], 2018, Perić et al. [33], 2018, Shirazi et al. [34], 2019, Lamichhane et al. [35], 2020, Souza e Silva et al. [36], 2020, Amarasinghe et al. [37], 2021 and Manikandan et al. [38], 2022. In contrast, 4 studies, such as Fatahinia et al. [23], 2015; Menezes et al. [24], 2015; Mun et al. [26], 2016; and Goulart et al. [32], 2018 presented a low risk of bias, with all criteria clearly met.

For the cohort studies, 5 studies, Sánchez-Vargas et al. [39], 2013; Sharma et al. [40], 2017; Silva et al. [41], 2018; Hu et al. [42], 2019; and Molkenthin et al. [43], 2022 showed a moderate risk of bias due to unclear or incomplete reporting on group similarity, confounding factors, follow-up, and statistical analysis. For the case-control study, Zomorodian et al. [44], 2016 also had a moderate risk of bias.

In general, the evidence in this review exhibits a low to moderate risk of bias across the included studies. The quality assessments of the studies are summarized in Table 3, Table 4 and Table 5.

### 3.3. Characteristics of the Included Studies

For each eligible study included in this systematic review, we extracted data on general characteristics, including author, year of publication, study design, objectives, study population, country, *Candida* spp., and key findings, as summarized in Table 6.

### 3.4. Quantitative and Geographic Distribution of Candida spp.

The total number of isolates across the 24 studies was 12,750. As shown in Table 7, *C. albicans* was the most prevalent species, accounting for 81.7% of all isolates. NAC species, including *C. tropicalis* (7.2%), *C. glabrata* (4.5%), *C. krusei* (4.1%), *C. parapsilosis* (1.0%), *C. dubliniensis* (0.8%), *C. kefyr* (0.2%), *C. guilliermondii* (0.1%), *C. lusitaniae* (0.1%), and other rare or unidentified species (0.2%), were observed at lower frequencies. The “Total Isolates” column represents both pure isolates and those found in combination with other species, providing a comprehensive overview of *Candida* distribution. These results emphasize the predominance of *C. albicans* while highlighting the clinical importance of NAC species in oral colonization and potential infections.

Regarding geographic distribution (Table 8), *C. albicans* was the predominant species across all continents, although its relative proportion varied considerably. The highest prevalence of *C. albicans* was observed in Asia, whereas North and South America exhibited a more balanced distribution between *C. albicans* and non-*albicans Candida* (NAC) species. Europe and Oceania showed intermediate patterns, with *C. albicans* remaining dominant but with a substantial contribution from NAC species. These geographic differences suggest that regional, environmental, and population-related factors may influence the distribution of *Candida* species, highlighting the importance of geographic context in epidemiological and clinical interpretations.

## 4. Discussion

### 4.1. Oral Colonization and Risk Factors for Candida Infections

Infections caused by *Candida* spp. have increased dramatically in recent decades, mainly due to the growing population of immunocompromised individuals [45,46,47]. The oral cavity hosts approximately 700 microbial species, including 20 *Candida* spp. While *Candida* is commensal in healthy individuals (35–70% prevalence), it can become opportunistic, particularly with the emergence of NAC species and rising antifungal resistance [23,25,27,29,30,34,38,39,41,48].

Oral colonization begins within hours of birth, peaking in the first month and stabilizing during the first year. Influencing factors include feeding type (breast milk or formula), maternal oral and vaginal microbiota, physiological changes with age, tooth eruption, environmental changes, diet, pacifier use, and daily habits [39,41]. *C. albicans* and other NAC species are increasingly reported, particularly among immunocompromised, diabetic, oncology, denture-wearing, or smoking patients, as well as those with salivary alterations or periodontal disease [21,26,27,29,38,39,41,42,43].

Smoking and active dental caries significantly increase the risk of oral candidiasis, with smokers being nearly seven times more likely to develop infection. Smoking promotes oral *Candida* colonization by altering the oral epithelium, reducing salivary flow and pH, decreasing leukocyte and immunoglobulin levels, and providing nutrients for fungal growth. In addition, it contributes to the production of carcinogenic compounds [26,30,33,43]. Oral candidiasis is also highly relevant in oncology patients, as approximately 70% experience treatment-related oral complications (mucositis, xerostomia) that favor *Candida* colonization [26,36]. Early monitoring, preventive measures, and timely treatment are essential to reduce complications and preserve both oral and systemic health.

### 4.2. Distribution in High-Risk Populations

#### 4.2.1. HIV-Positive Individuals

HIV infection is characterized by a reduced CD4+ T-lymphocyte count and an increased susceptibility to opportunistic infections. Oral candidiasis is often one of the earliest clinical indicators of HIV infection and occurs in approximately 35–95% of seropositive individuals, typically developing at least once during the course of the disease. The pseudomembranous form is particularly associated with advanced immunosuppression and carries prognostic significance, as its presence is frequently linked to progression toward AIDS [32,35,49].

In studies of oral *Candida* colonization among HIV-positive individuals, *C. albicans* is consistently the predominant species, commonly found in single-species colonization, but NAC species such as *C. glabrata*, *C. tropicalis*, and *C. parapsilosis* also contribute significantly. Goulart et al. [32] identified *C. glabrata* as the most frequent NAC species, while Spalanzani et al. [31] and Lamichhane et al. [35] reported *C. tropicalis* and *C. parapsilosis* as dominant, respectively.

Lourenço et al. [28] observed *C. albicans* and *C. parapsilosis* across all study groups, including HIV-negative and HIV-positive individuals, regardless of periodontal health. In contrast, *C. tropicalis* was restricted to HIV-positive patients, and *C. dubliniensis*, *C. glabrata*, and *C. krusei* were detected only in HIV-positive individuals with periodontal disease. Periodontal disease did not affect the presence or absence of *Candida* spp., but it increased fungal load by approximately 4.8-fold. Menezes et al. [24] similarly highlighted the clinical significance of mixed-species colonization, noting that *C. albicans* predominates in single-species and mixed-species colonization, while low CD4+ T-cell counts, antibiotic use, and oral prostheses are associated with higher fungal burden. Together, these findings indicate that immunosuppression and periodontal disease synergistically promote colonization by less common species, mixed-species biofilm formation, and increased risk of oral candidiasis.

The prevalence of oral candidiasis in HIV-positive patients varies geographically. In Brazil, studies have reported prevalences of 51.3% [32], 60.5% [24], and 73% [28]. In Asia, prevalence was 23.6% in Nepal [35], 49.5% in China [50], and 51.4% in Taiwan [51,52]. In sub-Saharan Africa, a review by Mushi MF. et al. [53] analyzed countries such as Nigeria, South Africa, Ethiopia, Uganda, Cameroon, Tanzania, and Ghana, reporting an overall prevalence of 50.6%, ranging from 7.6% in South Africa to 75.3% in Ghana. Another study in Côte d’Ivoire reported 79.4% [54]. In Europe, a study conducted in Spain reported a prevalence of 49.1% among HIV-positive patients [52]. Similarly, in Turkey, 82.8% of HIV-positive patients were found to have oral *Candida* colonization, with *C. albicans* being the most prevalent species, accounting for 83% of isolates [55]. These differences likely reflect variations in immunosuppression, oral hygiene, access to antiretroviral therapy, and local epidemiological factors.

Although *C. albicans* remains the most frequently isolated species, NAC species constitute a substantial proportion of isolates. Oral lesions caused by *Candida* spp., particularly pseudomembranous forms, serve as indirect markers of immunosuppression in HIV-positive individuals. Furthermore, the formation of mixed-species biofilms may contribute to antifungal resistance and persistence of chronic infection. Despite these findings, large and recent epidemiological studies in Europe are lacking, especially compared to Africa, Asia, and America.

#### 4.2.2. Diabetic Individuals

Diabetic individuals have a high predisposition to oral diseases, mainly due to poor glycemic control, which can contribute to xerostomia resulting from increased glucose levels in oral fluids or immune dysregulation. In addition, diabetes is associated with reduced salivary pH and a higher susceptibility to dental caries and periodontal disease. The increase in salivary glucose concentration enhances hemolysin production and reduces phagocytic activity due to impaired polymorphonuclear leukocytes, facilitating the adhesion and colonization of *Candida* spp. in the oral cavity of diabetic patients [21,23,27,39].

These factors promote adhesion, colonization, and biofilm formation by *Candida* spp. Mohammadi et al. [27] detected oral *Candida* colonization in 55% of diabetic individuals, predominantly *C. albicans*, followed by *C. krusei*, *C. glabrata*, and *C. tropicalis*. In non-diabetic individuals, only *C. albicans*, *C. krusei*, and *C. kefyr* were detected. Mixed-species biofilms were also observed exclusively in diabetic patients. Similarly, Zomorodian et al. [44] found *C. albicans* to be the most common species, with *C. dubliniensis* and *C. glabrata* representing important NAC isolates. Due to phenotypic similarities, *C. dubliniensis* is often misidentified as *C. albicans* when CHROM agar *Candida* is used, highlighting the need for molecular diagnostic [44].

Fatahinia et al. [23] reported a predominance of *C. glabrata* (14.3%) and *C. dubliniensis* (12.7%) among diabetic patients, whereas Sharma et al. [40] identified *C. parapsilosis* as the main NAC species (31.22%), surpassing *C. albicans* (28.01%), suggesting possible regional variation. Collectively, these findings indicate that diabetic patients not only exhibit higher colonization rates but also harbor a broader diversity of *Candida* spp., many of which demonstrate antifungal resistance.

#### 4.2.3. Denture Wearers

Microorganisms in the oral cavity that colonize denture surfaces form an adherent biofilm influenced by the prosthesis characteristics, oral hygiene practices, denture-cleaning agents, and the risk of self-inoculation or cross-infection during handling. Dentures made from synthetic polymers, such as polymethyl methacrylate, are microporous, facilitating *Candida* adhesion and colonization. More hydrophobic species, such as *C. glabrata*, adhere more strongly to acrylic surfaces than *C. albicans*, contributing to resilient biofilm formation. Dentures provide a reservoir for *Candida* spp., inducing palate changes, modifying saliva and oral microbiota, reducing salivary pH, and facilitating deposition of phosphate, calcium, and proteins on acrylic surfaces [25,33,37,56].

Over time, dentures gradually lose retention and stability due to inadequate occlusion and reduced vertical dimension. Older prostheses are more difficult to maintain because of increased porosities in the denture base, and surface roughness further enhances microbial adhesion. These factors collectively facilitate the adhesion, colonization, and biofilm maturation of *Candida* spp., reinforcing the denture as a niche for persistent oral colonization and potential infection [25,33,37].

Perić et al. [33] observed that individuals colonized by NAC species exhibited a higher degree of oral inflammation. Notably, *C. albicans* was the most prevalent species, followed by mixed-species biofilms comprising *C. albicans* and NAC species. Similarly, other studies reported *C. albicans* as the most prevalent species. However, its prevalence was higher among individuals without dentures compared to denture wearers, with *C. albicans* detected in 96.2% versus 58% in the study by Prakash et al. [25], and 78.3% versus 76.3% in the study by Amarasinghe et al. [37].

### 4.3. Overview of the Most Prevalent Candida spp.

The biofilm-forming capacity of NAC species has been reported to be higher than that of *C. albicans*, making biofilm formation a critical virulence factor that prolongs colonization and enables evasion of host defenses. This increased adherence is partly explained by their relative surface free energy values [22,33].

Analysis of the selected studies revealed that the most prevalent *Candida* spp. in oral candidiasis were *C. albicans* (81.7%), *C. tropicalis* (7.2%), *C. glabrata* (4.5%), *C. krusei* (4.1%), *C. parapsilosis* (1.0%), *C. dubliniensis* (0.8%), *C. kefyr* (0.2%), *C. guilliermondii* (0.1%), *C. lusitaniae* (0.1%), and other rare or unidentified species (0.2%) (Table 7). The studies originated primarily from Europe, Latin America, and Asia, with varying prevalence of NAC species across regions, possibly due to environmental and lifestyle factors.

*C. albicans* is the most common species in the oral cavity, affecting both young and adult individuals due to its strong adhesion and high pathogenic potential. As a dimorphic yeast, it can exist in yeast or hyphal forms depending on environmental conditions. It has been isolated from over 80% of oral lesions and is prevalent in 70–82% of individuals with AIDS [32,41]. Adhesion to hydroxyapatite is facilitated by collagenolytic enzymes that degrade dentin collagen and support fungal proliferation. Optimal enzyme activity occurs at pH 3.5–4.0, and lactic acid production through carbohydrate fermentation further weakens hydroxyapatite, contributing to lower salivary pH [27].

*C. tropicalis* exhibits higher virulence than *C. albicans*, leading to more aggressive infections and increased antifungal resistance. Its biofilm consists of blastoconidia and hyphae embedded in a thick extracellular polymeric matrix [21,22,33].

*C. glabrata* forms low-biomass, non-dimorphic biofilms composed of yeast cells within a thin extracellular matrix. It exhibits reduced susceptibility to common antifungals, making infections more severe and treatment-resistant compared to those caused solely by *C. albicans* [22,32,42]. Mixed biofilms of *C. glabrata* and *C. albicans* are associated with high levels of inflammation in denture stomatitis and display a synergistic effect in adhesion and colonization [22,48,54].

*C. krusei* produces thick biofilms and demonstrates inhibitory and antagonistic interactions with *C. albicans*, resulting in reduced mixed biofilm counts and decreased expression of genes related to adhesion and biofilm regulation [29,48].

*C. parapsilosis* forms compact, multilayered biofilms with moderate to strong biofilm-forming capacity, correlating with higher microbial load, and is associated with significant morbidity due to its pathogenic potential [22,29,40].

*C. dubliniensis* is often misidentified as *C. albicans* on CHROM agar *Candida* due to phenotypic similarities, including green colony formation, germ tube and chlamydospore production, and similar carbohydrate assimilation patterns. Molecular techniques such as PCR-RFLP using the AvrII restriction enzyme are effective for accurate differentiation [22,27,44]. Despite these similarities, *C. dubliniensis* is less pathogenic, less frequently isolated, and significantly associated with smoking and HIV-positive patients [43,44]. Expanding molecular diagnostics in routine clinical practice would enhance epidemiological accuracy and guide more effective antifungal stewardship.

### 4.4. Therapeutic Challenges and Resistance Patterns

Azole antifungals, particularly fluconazole, remain the most frequently prescribed agents for *Candida* infections. Azoles act by binding to and inhibiting the intracellular enzyme ERG11p, a key component of ergosterol biosynthesis [36,41]. Accurate species identification is clinically important because several *Candida* spp., including *C. glabrata*, *C. krusei*, and *C. tropicalis*, show reduced susceptibility or intrinsic resistance to azoles. Fluconazole resistance can be readily induced in vitro in *C. dubliniensis*, and exposure to this drug has been shown to increase its epithelial adhesion [27,31,33,36,43,44,57]. Rising resistance in *Candida* is associated with prolonged or repeated azole therapy and with high fungal cell densities [33,35,36,57].

Zomorodian et al. [44], Goulart et al. [32] reported resistance of *C. albicans* to fluconazole, ketoconazole, and itraconazole, whereas Lamichhane et al. [35], found that *C. albicans* remained highly susceptible to clotrimazole and fluconazole. However, the overall rates of azole resistance in isolates from HIV-positive patients were low. Furthermore, Perić et al. [33] observed that *C. tropicalis* exhibited greater resistance to antifungal agents compared to *C. albicans*.

Polyene antifungals such as nystatin and amphotericin B are generally recommended as first-line therapy for oral candidiasis. Nystatin targets ergosterol-containing fungal membranes, though its oral suspension may cause gastrointestinal discomfort, impaired glucose tolerance, and an increased risk of caries. Amphotericin B exerts fungicidal activity by binding membrane ergosterol and forming pores that lead to cell death; resistance due to altered ergosterol binding has been reported but remains rare. Amphotericin B is particularly relevant in invasive oral candidiasis or in cases of fluconazole resistance [41]. A study reported amphotericin B as the most effective antifungal overall, with clotrimazole demonstrating the highest activity against *Candida*, possibly reflecting widespread empirical use of amphotericin B without susceptibility testing [35]. Subtherapeutic concentrations of cetylpyridinium chloride and chlorhexidine digluconate have been shown to inhibit *Candida* spp. by reducing epithelial adhesion [36].

Miltefosine, an alkylphosphocholine originally developed as an antitumor agent, exhibits potent antiparasitic activity and inhibits *Candida* biofilm formation. Although oral miltefosine alone may not eradicate established biofilms, it shows promise as an adjuvant or pretreatment agent, particularly against fluconazole-resistant *Candida* biofilms [57].

### 4.5. Limitations

Heterogeneity in study populations, identification methods, and sample types likely influenced the reported *Candida* spp. and antifungal resistance. Differences among HIV-positive individuals, diabetic patients, children, oncology patients, and denture wearers, along with methods ranging from CHROM agar to PCR, affect detection accuracy. Sample type—saliva, smears, or lesions—also impacts isolation rates, while regional variations in *Candida* prevalence and antifungal use may further contribute to variability, limiting comparability across studies.

### 4.6. Clinical Implications and Future Perspectives

This review included studies involving HIV-positive individuals, diabetic patients, denture wearers, and the general population, highlighting the increased risk in these groups. Accurate species identification is essential, as NAC species differ in virulence and antifungal susceptibility and can affect treatment outcomes. Preventive measures, including improved oral hygiene, systemic disease control, and regular denture maintenance, remain crucial to reduce recurrence. Optimizing the use of existing antifungal therapies, together with strategies that limit biofilm formation, is important for managing resistant *Candida* infections.

## 5. Conclusions

The rising incidence of NAC species in oral candidiasis underscores the need for accurate identification to guide treatment. Although NAC species are increasingly detected, *C. albicans* remains by far the most prevalent species in the oral cavity. Key virulence factors, including strong adhesion, production of hydrolytic enzymes, dimorphism, phenotypic switching, and biofilm formation, significantly increase pathogenic potential, especially in immunocompromised individuals.

The limited antifungal options and current drug mechanisms contribute to growing resistance among NAC species. *C. krusei* and *C. glabrata* can disrupt *C. albicans* adhesion and biofilm development, suggesting competitive interactions during mixed-species biofilms. Most studies include samples from Europe, Latin America, and Asia, highlighting the need for broader global sampling. The most prevalent NAC species identified were *C. glabrata*, *C. tropicalis*, *C. parapsilosis*, *C. dubliniensis*, and *C. krusei*, especially among people living with HIV, diabetic patients, and denture wearers. Mixed-species biofilms are also becoming more common.

Therefore, considering the growing prevalence of antifungal-resistant *Candida* spp. and the competitive or synergistic interactions among them, the development of new therapeutic approaches is urgently needed.

## Figures and Tables

**Table 1:** PICOS Strategy.

P	Individuals diagnosed with oral candidiasis.
I	Identification and characterization of different *Candida* spp. involved in oral infections.
C	Comparison between *C. albicans* and non-*albicans Candida* spp. in terms of prevalence and distribution.
O	Determination of the most prevalent *Candida* spp. responsible for oral candidiasis.

**Table 2:** Databases, research strategy and articles retrieved.

Databases	Advanced Research	Articles Found
PubMed	(*Candida* spp. AND Oral Candidiasis AND Oral isolates)	159
Cochrane Library	*Candida* spp. AND Oral Candidiasis AND Oral isolates	5
ScienceDirect	*Candida* spp. AND Oral Candidiasis AND Oral isolates	521

**Table 3:** Joanna Briggs Institute Critical Appraisal Checklist for Cross Sectional Studies.

	Were the Criteria for Inclusion in the Sample Clearly Defined?	Were the Study Subjects and the Setting Described in Detail?	Was the Exposure Measured in a Valid and Reliable Way?	Were Objective, Standard Criteria Used for Measurement of the Condition?	Were Confounding Factors Identified?	Were Strategies to Deal with Confounding Factors Stated?	Were the Outcomes Measured in a Valid and Reliable Way?	Was Appropriate Statistical Analysis Used?
Sanitá et al. [21], 2014	U	Y	U	Y	Y	N	N	Y
Muadcheingka et al. [22], 2015	U	Y	U	Y	Y	N	N	Y
Fatahinia et al. [23], 2015	Y	Y	Y	Y	Y	Y	Y	Y
Menezes et al. [24], 2015	Y	Y	Y	Y	Y	Y	Y	Y
Prakash et al., 2015 [25]	Y	Y	Y	Y	Y	U	N	Y
Mun et al. [26], 2016	Y	Y	Y	Y	Y	Y	Y	Y
Mohammadi et al. [27], 2016	Y	Y	Y	Y	Y	Y	U	Y
Lourenço et al. [28], 2017	Y	Y	Y	Y	Y	Y	U	Y
Portela et al. [29], 2017	Y	Y	Y	Y	Y	U	U	Y
Castillo et al. [30], 2018	U	Y	U	Y	Y	U	N	Y
Spalanzani et al. [31], 2018	Y	Y	Y	Y	Y	U	N	Y
Goulart et al. [32], 2018	Y	Y	Y	Y	Y	Y	Y	Y
Perić et al. [33], 2018	Y	Y	Y	Y	Y	Y	U	Y
Shirazi et al. [34], 2019	U	Y	U	Y	Y	U	N	Y
Lamichhane et al. [35], 2020	Y	Y	Y	Y	Y	Y	U	Y
Souza e Silva et al. [36], 2020	Y	Y	Y	Y	Y	U	N	Y
Amarasinghe et al. [37], 2021	Y	Y	Y	Y	Y	U	N	Y
Manikandan et al., 2022 [38]	Y	Y	Y	Y	Y	U	N	Y

(Y)—Yes, (N)—No, (U)—Unclear.

**Table 4:** Joanna Briggs Institute Critical Appraisal Checklist for Cohort Studies.

	Were the Two Groups Similar and Recruited from the Same Population?	Were the Exposures Measured Similarly to Assign People to Both Exposed and Unexposed Groups?	Was the Exposure Measured in a Valid and Reliable Way?	Were Confounding Factors Identified?	Were Strategies to Deal with Confounding Factors Stated?	Were the Groups/Participants Free of the Outcome at the Start of the Study (or at the Moment of Exposure)?	Were the Outcomes Measured in a Valid and Reliable Way?	Was the Follow Up Time Reported and Sufficient to Be Long Enough for Outcomes to Occur?	Was Follow Up Complete, and If Not, Were the Reasons to Loss to Follow Up Described and Explored?	Were Strategies to Address Incomplete Follow Up Utilized?	Was Appropriate Statistical Analysis Used?
Sánchez-Vargas et al. [39], 2013	U	Y	Y	U	N	Y	Y	Y	N	N	Y
Sharma et al. [40], 2017	U	Y	Y	U	N	Y	Y	U	U	N	Y
Silva et al. [41], 2018	Y	Y	Y	U	N	Y	Y	Y	N	N	Y
Hu et al. [42], 2019	Y	Y	U	N	N	U	Y	Y	U	N	Y
Molkenthin et al. [43], 2022	U	U	Y	Y	U	U	Y	Y	U	N	Y

(Y)—Yes, (N)—No, (U)—Unclear.

**Table 5:** Joanna Briggs Institute Critical Appraisal Checklist for Case Control Studies.

	Were the Groups Comparable Other Than the Presence of Disease in Cases or the Absence of Disease in Controls?	Were Cases and Controls Matched Appropriately?	Were the Same Criteria Used for Identification of Cases and Controls?	Was Exposure Measured in a Standard, Valid and Reliable Way?	Was Exposure Measured in the Same Way for Cases and Controls?	Were Confounding Factors Identified?	Were Strategies to Deal with Confounding Factors Stated?	Were Outcomes Assessed in a Standard, Valid and Reliable Way for Cases and Controls?	Was the Exposure Period of Interest Long Enough to Be Meaningful?	Was Appropriate Statistical Analysis Used?
Zomorodian et al. [44], 2016	U	N	Y	Y	Y	U	N	Y	Y	Y

(Y)—Yes, (N)—No, (U)—Unclear.

**Table 6:** Descriptive analysis of selected scientific articles.

Authors and Year of Publication	Study Design	Objectives	Study Population	Country	*Candida* spp.	Key Findings
Sánchez-Vargas et al. [39], 2013	Longitudinal observational Study	To evaluate the influence of predisposing factors on colonization, infection, and biofilm formation kinetics.	*n* = 63 individuals	Mexico	*C. albicans* (*n* = 39) *C. tropicalis* (*n* = 15) *C. glabrata* (*n* = 7) *C. krusei* (*n* = 4) *C. lusitaniae* (*n* = 1) *C. kefyr* (*n* = 1) *C. guilliermondii* (*n* = 1) *C. pulcherrima* (*n* = 1)	- Oral isolates of *C. glabrata* were strong biofilm producers, while *C. albicans* and *C. tropicalis* were moderate. - Biofilm kinetics varied by strain origin and metabolic profile.
Sanitá et al. [21], 2014	Cross-sectional observational study	To assess phospholipase (PL) and secreted aspartyl proteinase (SAP) expression in *C. glabrata* and *C. tropicalis*.	*n* = 51 individuals (16 healthy, 10 diabetic with oral candidiasis, 25 non-diabetic with oral candidiasis)	Brazil	*C. tropicalis* (*n* = 20) *C. glabrata* (*n* = 31)	- NAC species also exhibit the ability to secrete secreted aspartyl proteinases (SAPs) and phospholipases (PLs) in vitro. - *C. tropicalis* showed higher secretion rates of these enzymes compared to *C. glabrata*. - These virulence mechanisms enable yeasts to colonize oral and mucosal surfaces, invade deeper host tissues, evade host immune defenses, and cause infections. - The secretion of SAPs and PLs by different *Candida* spp. may represent a potential target for antifungal therapy.
Muadcheingka et al. [22], 2015	Cross-sectional observational study	To determine the prevalence of *C. albicans* and NAC species and evaluate cell surface hydrophobicity (CSH) and biofilm-forming ability.	*n* = 207 patients with oral candidiasis (with/without denture)	Thailand	*C. albicans* (*n* = 154) *C. glabrata* (*n* = 38) *C. tropicalis* (*n* = 26) *C. kefyr* (*n* = 9) *C. parapsilosis* (*n* = 8) *C. lusitaniae* (*n* = 5) *C. dubliniensis* (*n* = 5) *C. krusei* (*n* = 4) *C. guilliermondii* (*n* = 1)	- *C. albicans* was the most prevalent species (61.6%) in patients with oral candidiasis, both with and without dentures. - NAC species included: *C. glabrata* (15.2%), *C. tropicalis* (10.4%), *C. parapsilosis* (3.2%), *C. kefyr* (3.6%) Others (<2%) - NAC species had higher biofilm biomass and cell surface hydrophobicity than *C. albicans*. Biofilm formation: 92% of NAC isolates formed biofilm 78% of *C. albicans* isolates formed biofilm. - Gradual increase in colonization by NAC species may be due to their enhanced biofilm formation and hydrophobicity.
Fatahinia et al. [23], 2015	Cross-sectional observational study	Compare esterase and hemolytic activity in various *Candida* spp. isolated from the oral cavity of diabetic and non-diabetic individuals.	*n* = 190 patients (95 diabetics; 95 healthy controls)	Iran	***Diabetic group:*** *C. albicans* (*n* = 42) *C. dubliniensis* (*n* = 8) *C. krusei* (*n* = 9) *C. glabrata* (*n* = 4) ***Non-diabetic group:*** *C. albicans* (*n* = 16) *C. dubliniensis* (*n* = 4) *C. krusei* (*n* = 4) *C. glabrata* (*n* = 4)	- Esterase activity was detected in all *Candida* isolates. - Hemolytic activity was higher in the diabetic group compared to the non-diabetic group. - Only 21.6% of diabetic patients showed esterase activity. - Hemolytic activity was observed in *C. albicans*, *C. dubliniensis*, *C. glabrata*, and *C. krusei*.
Menezes et al. [24], 2015	Cross-sectional observational study	To assess colonization and quantify *Candida* spp. in the oral cavity, determine predisposing factors for colonization, and correlate CD4+ cell levels and viral load in HIV-positive patients.	*n* = 147 HIV-infected patients	Brazil	*C. albicans* (*n* = 94) *C. parapsilosis* (*n* = 17) *C. tropicalis* (*n* = 15) *C. glabrata* (*n* = 8) *C. krusei* (*n* = 6) *C. dubliniensis* (*n* = 5) *C. kefyr* (*n* = 3) *C. famata* (*n* = 1) *C. guilliermondii* (*n* = 1) *C. lusitaniae* (*n* = 2) *C. pelliculosa* (*n* = 1)	- *C. albicans* was the most frequent species (67.6%) in single-species colonization and was also frequently found in association with other *Candida* spp. in mixed-species colonization. - The main predisposing factors for oral *Candida* colonization were antibiotic use and oral prostheses. - Reverse transcriptase inhibitor therapy appeared to have a protective effect against colonization. - Low CD4+ T-cell counts were associated with higher yeast density in the saliva of HIV-positive patients.
Prakash et al. [25], 2015	Cross-sectional observational study	To assess the prevalence of *Candida* spp. in denture-wearing individuals and healthy non-denture-wearing individuals, in relation to age and oral hygiene status.	*n* = 50 Denture wearers (DW) *n* = 50 Non-denture wearers (NDW)	India	***DW group:*** *C. albicans* (*n* = 29) *C. tropicalis* (*n* = 14) *C. dubliniensis* (*n* = 6) *C. glabrata* (*n* = 1) ***NDW group:*** *C. albicans* (*n* = 25) *C. tropicalis* (*n* = 1)	- The prevalence of *Candida* spp. was higher in males than in females. - The prevalence of *C. albicans* increased with age in males: Highest in the 66–75 years age group (66.7%) Lowest in the 36–45 years age group (58.3%) In females, *C. albicans* prevalence: Highest in the 36–45 years age group (41.7%) Lowest in the 66–75 years age group (22.2%). - Oral hygiene influenced the prevalence of *Candida* spp. in both groups. - Males had poorer oral hygiene compared to females.
Mun et al. [26], 2016	Cross-sectional observational study	To analyze the prevalence of *Candida* in the oral cavity of non-oncological individuals, in relation to the presence of external factors affecting the oral environment.	*n* = 203 individuals	Australia	*C. albicans* (*n* = 82) *C. krusei* (*n* = 11) *NAC species* (*n* = 16)	- Both smoking and the presence of active carious lesions were positively correlated with *Candida* infection. - Smokers were nearly seven times more likely to have oral candidiasis. - *C. albicans* was the predominant species, carried by 84.7% of positive cases. - NAC species were present in a minority of cases, either alone or in mixed-species colonization. - Higher yeast loads were more frequently observed in current smokers.
Zomorodian et al. [44], 2016	Case-control Study	To evaluate oral *Candida* colonization in diabetic patients and its association with species, serum glucose, and antifungal susceptibility.	*n* = 113 patients with type 2 diabetes, 24 patients with type 1 diabetes, and 105 healthy controls	Iran	***Diabetic Group:*** *C. albicans* (*n* = 94) *C. dubliniensis* (*n* = 25) *C. glabrata* (*n* = 17) *C. parapsilosis* (*n* = 5) *C. guilliermondii* (*n* = 3) *C. krusei* (*n* = 2) *C. kefyr* (*n* = 2) *C. tropicalis* (*n* = 2) ***Non-Diabetic Group:*** *C. albicans* (*n* = 47) *C. dubliniensis* (*n* = 5) *C. glabrata* (*n* = 9) *C. guilliermondii* (*n* = 3) *C. parapsilosis* (*n* = 1) *Rhodotorula* (*n* = 1)	- *C. albicans* was the most prevalent species, including in mixed colonization. - Poor glycemic control was associated with higher *Candida* prevalence and density in diabetics. - Highest antifungal resistance was observed for itraconazole, followed by ketoconazole and fluconazole. - *C. dubliniensis* was frequently found in diabetics and may be misdiagnosed as *C. albicans.*
Mohammadi et al. [27], 2016	Cross-sectional observational study	To identify and compare the colonization level of *Candida* spp. in the oral cavity of diabetic and non-diabetic groups.	*n* = 58 diabetic patients and 47 non-diabetics patients	Iran	***Diabetic Group:*** *C. albicans* (*n* = 25) *C. krusei* (*n* = 6) *C. glabrata* (*n* = 3) *C. tropicalis* (*n* = 2) ***Non-Diabetic Group:*** *C. albicans* (*n* = 13) *C. krusei* (*n* = 2) *C. kefyr* (*n* = 2)	- The most frequent species were, respectively, *C. albicans*, *C. krusei*, *C. glabrata*, and *C. tropicalis*. - Xerostomia and alterations in physiological factors, such as salivary pH and glucose, can promote *Candida* overgrowth in the oral cavity. - These factors are important predisposing conditions for oral candidiasis in diabetic patients.
Lourenço et al. [28], 2017	Cross-sectional observational study	To investigate the effects of periodontal conditions on the prevalence of *Candida* spp. in HIV-infected and non-HIV-infected individuals.	*n* = 48 HIV-infected patients and 25 healthy patients	Brazil	***Group A—Non-HIV-infected, periodontally healthy*** *C. albicans* (*n* = 7) *C. parapsilosis* (*n* = 3) ***Group B—Non-HIV-infected, periodontally affected*** *C. albicans* (*n* = 8) *C. parapsilosis* (*n* = 2) ***Group C—HIV-infected, periodontally healthy*** *C. albicans* (*n* = 11) *C. parapsilosis* (*n* = 1) *C. tropicalis* (*n* = 1) ***Group D—HIV-infected, periodontally affected*** *C. albicans* (*n* = 23) *C. krusei* (*n* = 2) *C. parapsilosis* (*n* = 2) *C. tropicalis* (*n* = 2) *C. dubliniensis* (*n* = 1) *C. glabrata* (*n* = 3)	- HIV-infected patients with healthy periodontal conditions had similar *Candida* spp. levels to non-HIV-infected patients. - Periodontal disease in HIV-infected patients significantly increased *Candida* spp. counts. - In non-HIV-infected patients, periodontal status did not affect *Candida* spp. prevalence. - Periodontal disease may predispose HIV-infected patients to higher *Candida* carriage and candidiasis.
Portela et al. [29], 2017	Cross-sectional observational study	To assess the biofilm viability, as well as the phospholipase and protease production of *Candida* spp. from the saliva of HIV-infected children and healthy controls	*n* = 43 HIV infected children and 17 healthy children	Brazil	***HIV-infected group:*** *C. albicans* (*n* = 33) *C. parapsilosis* (*n* = 12) *C. krusei* (*n* = 8) *C. tropicalis* (*n* = 1) *C. dubliniensis* (*n* = 1) *C. guilliermondii* (*n* = 1) ***Healthy group:*** *C. albicans* (*n* = 15) *C. parapsilosis* (*n* = 8)	- Biofilm activity and viability were higher in isolates of *C. albicans* than in isolates of NAC. - Although *Candida* spp. isolates from HIV-positive children showed higher phospholipase production, in vitro they exhibited reduced virulence factors compared to isolates from healthy individuals. - This finding suggests that immunosuppression may play an important role in the development of *Candida* virulence.
Sharma et al. [40], 2017	Prospective observational study	To identify and compare different *Candida* spp. in the oral cavity of Type II diabetic individuals	*n* = 30 Type II diabetic patients and 30 healthy patients	India	***Diabetic Group:*** *C. parapsilosis* (*n* = 8) *C. albicans* (*n* = 8) *C. glabrata* (*n* = 3) *C. krusei* (*n* = 3) *C. dubliniensis* (*n* = 3) *C. tropicalis* (*n* = 2) ***Non-Diabetic Group:*** *C. parapsilosis* (*n* = 11) *C. albicans* (*n* = 8) *C. glabrata* (*n* = 3) *C. krusei* (*n* = 2) *C. dubliniensis* (*n* = 3) *C. tropicalis* (*n* = 3)	- *C. albicans*, *C. glabrata*, *C. dubliniensis*, *C. krusei*, and *C. parapsilosis* showed significantly higher occurrence in diabetic patients (except *C. tropicalis*). - *C. parapsilosis* was the most frequent species, followed by *C. albicans.* - The increased presence of *C. parapsilosis* indicates a higher risk of oral infection, with limited treatment options.
Castillo et al. [30], 2018	Cross-sectional observational study	To analyze the association between malignant and pre-malignant oral lesions and the virulence factor profile of *Candida* spp.	*n* = 25 chronic oral candidiasis; 11 atypical lichen planus; 25 Oral squamous cell carcinoma; 15 Asymptomatic healthy individuals	Argentina	*C. albicans* (*n* = 33) *C. krusei* (*n* = 11) *C. tropicalis* (*n* = 11) *C. dubliniensis* (*n* = 2) *C. glabrata* (*n* = 2)	- NAC species showed higher biofilm formation than *C. albicans*. - No significant differences were observed in virulence factors between species. - A strong positive correlation was found between proteinase and lipase activity, and between hydrophobicity and biofilm formation, providing evidence of the association between *Candida* pathogenicity and lesion severity.
Spalanzani et al. [31], 2018	Cross-sectional observational study	To evaluate the correlation between the presence of oral lesions caused by *Candida* spp. in HIV-positive patients.	*n* = 66 HIV-infected patients	Brazil	*C. albicans* (*n* = 30) *C. tropicalis* (*n* = 6) *C. krusei* (*n* = 4) *C. parapsilosis* (*n* = 2) *C. glabrata* (*n* = 2) *C. dubliniensis* (*n* = 1)	- Oral lesions, mainly pseudomembranous, were associated with higher levels of immunosuppression. - *Candida* spp. were frequently detected in HIV-positive patients, both with and without oral lesions. - Lower antifungal susceptibility among NAC isolates highlights the importance of susceptibility testing to guide treatment.
Goulart et al. [32], 2018	Cross-sectional observational study	To analyze antifungal susceptibility and factors associated with oral colonization by *Candida* spp. in HIV-positive individuals.	*n* = 197 HIV-infected patients	Brazil	*C. albicans* (*n* = 81) *C. glabrata* (*n* = 14) *C. tropicalis* (*n* = 4) *C. krusei* (*n* = 2)	- *C. albicans* was the most prevalent species (80%). - Age (45 years or older) was the only factor associated with oral colonization by *Candida* spp. - Low rates of antifungal resistance to azoles were detected in yeast isolates from HIV-positive patients. - Resistance to fluconazole, ketoconazole, and itraconazole was 1%, 1%, and 4%, respectively.
Perić et al. [33], 2018	Cross-sectional observational study	To study possible risk factors associated with denture-induced stomatitis (DIS), assess its severity according to Newton’s classification, and investigate the association with the presence of NAC.	*n* = 113 patients with DIS	Serbia	*C. albicans* (*n* = 47) *C. krusei* (*n* = 17) *C. tropicalis* (*n* = 13) *C. glabrata* (*n* = 12)	- The higher adhesion of some NAC species (*C. tropicalis*, *C. glabrata*, and *C. krusei*) is explained by their relative surface free energy values. - Similarly, more hydrophobic microorganisms, such as *C. glabrata*, appear to adhere better to acrylic surfaces than *C. albicans*. - *C. tropicalis* showed greater resistance to antifungal agents compared to *C. albicans*.
Silva et al. [41], 2018	Longitudinal observational study	To evaluate the incidence of *Candida* spp. in groups of children with orofacial clefts during preoperative, postoperative, and up to the first follow-up visit. To assess in vitro antifungal susceptibility and virulence profiles.	*n* = 46 children with orofacial clefts	Brazil	***Period A—pre-asepsis*** *C. albicans* (*n* = 7) *C. krusei* (*n* = 4) *C. tropicalis* (*n* = 8) *C. krusei + C. tropicalis* (*n* = 1) ***Period B—post-asepsis*** *No species detected* ***Period C—post-surgery*** *C. albicans* (*n* = 4) *C. tropicalis* (*n* = 1) ***Period D—post-surgery follow-up*** *C. albicans* (*n* = 1) *C. krusei* (*n* = 5) *C. tropicalis* (*n* = 2) *C. albicans + C. tropicalis/C. albicans + C. krusei* (*n* = 2)	- Low incidence (39.1%) of oral colonization by *Candida* spp. was reported. - Significant reduction in *Candida* frequencies and species changes over the sampling periods indicated dynamic patterns of oral colonization: elimination, maintenance, or recolonization of the biotypes.
Hu et al. [42], 2019	Retrospective observational study	To assess the prevalence of oral candidiasis and the distribution of *Candida* spp. in patients with oral mucosal diseases.	*n* = 9769 patients	China	*C. albicans* (*n* = 8412) *C. tropicalis* (*n* = 676) *C. krusei (n =* 311) *C. glabrata* (*n =* 226)	- Over four years, *C. albicans* remained the most common species (75.37%), followed by *C. tropicalis* (6.06%), *C. krusei* (2.79%), and *C. glabrata* (2.02%). - Notably, both the proportion and number of *C. glabrata* isolates increased markedly over this period.
Shirazi et al. [34], 2019	Cross-sectional observational study	To evaluate the most prevalent *Candida* spp. in pediatric patients and the risk factors associated with oropharyngeal candidiasis.	*n* = 1152 children	Pakistan	*C. albicans (n =* 790) *C. glabrata (n =* 149) *C. krusei* (*n* = 98) *C. tropicalis* (*n* = 65) *C. parapsilosis* (*n* = 36) *C. dubliniensis* (*n* = 13) *C. lusitaniae* (*n* = 5)	- *C. albicans* was the most prevalent species (68.6%), while *C. lusitaniae* was the least prevalent (0.4%). - Associated risk factors included maternal hygiene, the patient’s oral hygiene, and the parents’ economic status. - Since it is a childhood disease, oropharyngeal candidiasis often presents with multiple recurrent episodes and, if not treated properly, can lead to severe invasive and non-invasive infections.
Lamichhane et al. [35], 2020	Cross-sectional observational study	To explore biofilm-producing *Candida* spp. causing oropharyngeal infections in HIV patients.	*n* = 174 HIV-infected patients	Nepal	*C. albicans* (*n* = 25) *C. parapsilosis* (*n* = 6) *C. dubliniensis* (*n* = 4) *C. tropicalis* (*n* = 3) *C. glabrata* (*n* = 2) *C. guillermondi* (*n* = 1)	- Oropharyngeal candidiasis is a common opportunistic infection in HIV-infected individuals, affecting 23.6% of patients. - 65% of the isolates were biofilm producers. - *C. albicans* showed high susceptibility to clotrimazole (96%) and fluconazole (92%). - Biofilm-producing isolates showed higher resistance to antifungal drugs, especially ketoconazole (51.9%).
Souza e Silva et al. [36], 2020	Cross-sectional observational study	To determine the prevalence of *Candida* spp. in cancer patients and to evaluate the antimicrobial activity of antiseptics in comparison with antifungal agents.	*n* = 34 cancer patients	Brazil	*C. albicans* (*n* = 7) *C. glabrata* (*n* = 3) *C. tropicalis* (*n* = 2)	- Patients with mucositis are at higher risk of oral colonization by *Candida* spp. - A high prevalence of mucositis and oral colonization by *Candida* spp. was observed in cancer patients. - Development of antifungal resistance may occur. The use of antiseptics contributed to maintaining oral health in these patients.
Amarasinghe et al. [37], 2021	Cross-sectional observational study	To identify *Candida* spp. in individuals with DIS and in healthy individuals, evaluating their production of invasive enzymes.	*n* = 38 individuals with DIS and 23 healthy individuals	Sri Lanka	***DIS group:*** *C. albicans* (*n* = 29) *C. glabrata* (*n* = 5) *C. parapsilosis* (*n* = 2) *C. tropicalis* (*n* = 2) ***Healthy group:*** *C. albicans* (*n* = 18) *C. parapsilosis* (*n* = 3) *C. glabrata* (*n* = 1) *C. guilliermondii* (*n* = 1)	- *C. albicans* genotype A was the most common (69%) in both pathogenic and commensal strains, followed by genotype C (20.7%) and genotype B (10.3%) in pathogenic isolates. - *C. albicans* from DIS patients exhibits significantly higher enzymatic activity (phospholipase, esterase, and hemolysin) compared to commensal isolates. - Increased enzymatic activity likely contributes to the pathogenicity of *C. albicans* in DIS.
Molkenthin et al. [43], 2022	Retrospective observational study	To identify factors associated with the presence of *C. dubliniensis* and other NAC species in oral lichen planus (OLP) lesions.	*n* = 268 individuals with OLP	Germany	*C. albicans* (*n* = 138) *C. glabrata* (*n* = 14) *C. dubliniensis* (*n* = 11) *C. krusei* (*n* = 5) *C. parapsilosis* (*n* = 5) *C. tropicalis* (*n* = 3) *C. guilliermondii* (*n* = 3) *C. kefyr* (*n* = 3) Other species (*n* = 9)	- *C. albicans* was the most frequently isolated species (72.3%), followed by *C. glabrata* (7.3%), *C. dubliniensis* (5.8%), and *C. krusei* and *C. parapsilosis* (both 2.6%). - The presence of *C. dubliniensis* was significantly associated with smoking. - Other NAC species were more frequently detected in patients using removable dentures. - In patients with OLP, certain local and systemic factors increase the risk of carrying potentially drug-resistant *Candida* spp. and developing *Candida* superinfection.
Manikandan et al. [38], 2022	Cross-sectional observational study	To compare *Candida* colonization in DW and NDW.	*n* = 30 complete DW and 30 NDW	India	*C. albicans* (*n* = 30) *C. tropicalis* (*n* = 20) *C. glabrata* (*n* = 11) *C. krusei* (*n* = 1)	- A significant association was observed between DW and the growth of *C. albicans* and *C. krusei.* - Denture material, duration of denture use, immunosuppression, and age are associated risk factors for the development of oral candidiasis.

*C. albicans*—*Candida albicans*; *C. dubliniensis*—*Candida dubliniensis*; *C. famata*—*Candida famata*; *C. glabrata*—*Candida glabrata*; *C. guilliermondii*—*Candida guilliermondii*; *C. kefyr*—*Candida kefyr*; *C. krusei*—*Candida krusei*; *C. lusitaniae*—*Candida lusitaniae*; *C. parapsilosis*—*Candida parapsilosis*; *C. pelliculosa*—*Candida pelliculosa*; *C. pulcherrima*—*Candida pulcherrima*; *C. tropicalis*—*Candida tropicalis*; CSH—Cell Surface Hydrophobicity; DIS—Denture-induced stomatitis; DW—Denture wearers; HIV—Human Immunodeficiency Virus; NDW—Non-denture wearers; OLP—Oral lichen planus; PL—Phospholipase; Rhodotorula—Rhodotorula spp.; SAP—Secreted Aspartyl Proteinase.

**Table 7:** Frequency and relative proportion of *Candida* spp. identified in the 24 included studies.

*Candida* spp.	Total Isolates (Isolated or Combined)	Percentage (%)
*C. albicans*	10,427	81.7
*C. tropicalis*	922	7.2
*C. glabrata*	572	4.5
*C. krusei*	523	4.1
*C. parapsilosis*	132	1.0
*C. dubliniensis*	97	0.8
*C. kefyr*	20	0.2
*C. guilliermondii*	15	0.1
*C. lusitaniae*	13	0.1
Other species	29	0.2

**Table 8:** Geographic distribution of *Candida* spp. by continent.

Continent	Countries Included	Number of Studies	*C. albicans n* (%)	NAC *n* (%)
North America	Mexico	1	39 (56.5%)	30 (43.5%)
South America	Brazil, Argentina	9	354 (58.9%)	247 (41.1%)
Asia	Iran, India, Pakistan, Nepal, Thailand, Sri Lanka, China	11	9765 (83.6%)	1923 (16.4%)
Europe	Germany, Serbia	2	185 (66.1%)	95 (33.9%)
Oceania	Australia	1	82 (75.2%)	27 (24.8%)

## Data Availability

No new data were created or analyzed in this study. Data sharing is not applicable to this article.

## Abbreviations

The following abbreviations are used in this manuscript:

*C. albicans*	*Candida albicans*
*C. dubliniensis*	*Candida dubliniensis*
*C. famata*	*Candida famata*
*C. glabrata*	*Candida glabrata*
*C. guilliermondii*	*Candida guilliermondii*
*C. kefyr*	*Candida kefyr*
*C. krusei*	*Candida krusei*
*C. lusitaniae*	*Candida lusitaniae*
*C. parapsilosis*	*Candida parapsilosis*
*C. pelliculosa*	*Candida pelliculosa*
*C. pulcherrima*	*Candida pulcherrima*
*C. tropicalis*	*Candida tropicalis*
*Candida* spp.	*Candida* species
CSH	Cell Surface Hydrophobicity
DIS	Denture-induced stomatitis
DW	Denture wearers
HIV	Human Immunodeficiency Virus
JBI	Joanna Briggs Institute
NAC	Non-*albicans Candida*
NDW	Non-denture wearers
OLP	Oral lichen planus
PL	Phospholipase
*Rhodotorula* spp.	*Rhodotorula* species
SAP	Secreted Aspartyl Proteinase
